# Case Report: von Hippel-Lindau (VHL) disease: a young female presenting with multiple organ tumors

**DOI:** 10.3389/fgene.2025.1676039

**Published:** 2025-10-17

**Authors:** Jingyuan Li, Yun Ti, Xiao Yang, Keqiang Yan, Tongshuai Chen, Tao Guo, Qin Hu, Cheng Zhang, Peili Bu

**Affiliations:** ^1^ State Key Laboratory for Innovation and Transformation of Luobing Theory, Key Laboratory of Cardiovascular Remodeling and Function Research of MOE, NHC, CAMS and Shandong Province, Department of Cardiology, Qilu Hospital of Shandong University, Jinan, China; ^2^ Department of Urology, Qilu Hospital, Shandong University, Jinan, China

**Keywords:** secondary hypertension, von Hippel-Lindau (VHL) disease, pheochromocytoma, spinal cord hemangioblastoma, multiple pancreatic cysts

## Abstract

This report presents a case involving a young female patient diagnosed with von Hippel-Lindau (VHL) syndrome. The patient developed multiple sequential tumors, including spinal cord hemangioblastoma, multiple pancreatic cysts, and pheochromocytoma. Whole-genome sequencing identified a deletion mutation in exon 3 of the VHL gene, challenging the previous understanding that VHL deletion mutations are highly prevalent in hemangioblastoma and renal cell carcinoma but uncommon in pheochromocytoma. This case underscores the importance of considering hereditary genetic syndromes in patients presenting with hypertension and multi-system tumor involvement. Comprehensive evaluation, standardized familial screening, and genetic testing play a crucial role in guiding treatment decisions for affected individuals.

## History of presentation

A 23-year-old female patient presented to the ophthalmology department with a chief complaint of blurred vision in the right eye accompanied by dizziness for 1 week. Fundus photography and optical coherence tomography revealed hypertensive retinopathy with macular edema in the right eye and optic neuropathy in the left eye. Ophthalmologists recognize that retinopathy may be associated with hypertension and routinely recommend blood pressure monitoring for the patient. The patient had no prior history of blood pressure measurement; however, during a visit to the ophthalmology outpatient clinic, her blood pressure was recorded at 200/120 mmHg, indicating severe hypertension. Following admission to the hypertension ward, the medical team initiated active blood pressure management and conducted a comprehensive evaluation to identify potential secondary causes of hypertension. Physical examination, electrocardiogram and echocardiography did not reveal any abnormal signs. Computed tomography (CT) imaging demonstrated that: (1) A mass measuring approximately 5.9 × 4.9 cm in maximum cross-sectional area is observed in the right adrenal region, with indistinct demarcation from the adrenal gland and central patchy necrosis. (2) The pancreas is enlarged and shows multiple diffusely distributed cystic low-density lesions, consistent with multiple pancreatic cysts. (3) No significant stenosis or dilatation is identified in the bilateral renal arteries.

## Past medical history

**FIGURE 1:**
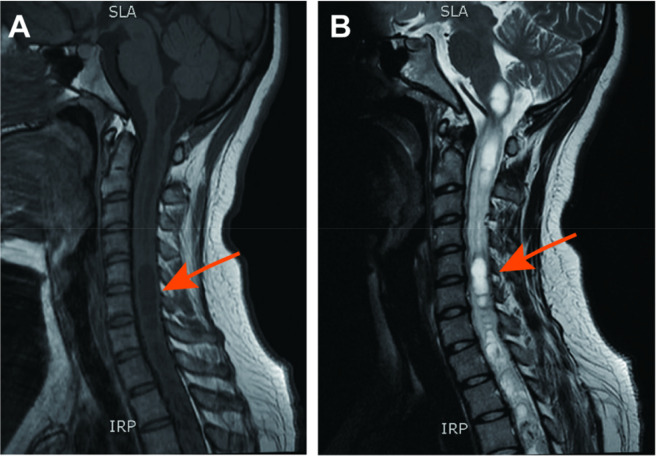
Spinal cord magnetic resonance imaging in 2020 showing a space-occupying lesion within the spinal canal at the T2-T5 level. **(A)** T1-weighted magnetic resonance image. **(B)** T2-weighted magnetic resonance image.

Four years ago, the patient presented to the neurology department with progressive weakness and reduced sensation in the right lower limb. Magnetic resonance imaging (MRI) revealed a space-occupying lesion within the spinal canal at the T2-T5 level, accompanied by syringomyelia formation above and below the lesion (Figure 1). Postoperative pathological analysis confirmed the diagnosis of spinal cord hemangioblastoma. Currently, muscle strength and sensory function in both lower limbs are within normal limits.

## Differential diagnosis

The differential diagnosis of adrenal tumors includes aldosteronoma, cortisoloma and pheochromocytoma, as well as adrenal cortical carcinoma and metastatic lesions. Other considerations include adrenal cysts and hemorrhage. Given the patient’s age, along with a history of hemangioblastoma and hypertension, the clinical evaluation should prioritize functional adrenal tumors, VHL syndrome and potential metastatic disease.

## Investigations

**FIGURE 2:**
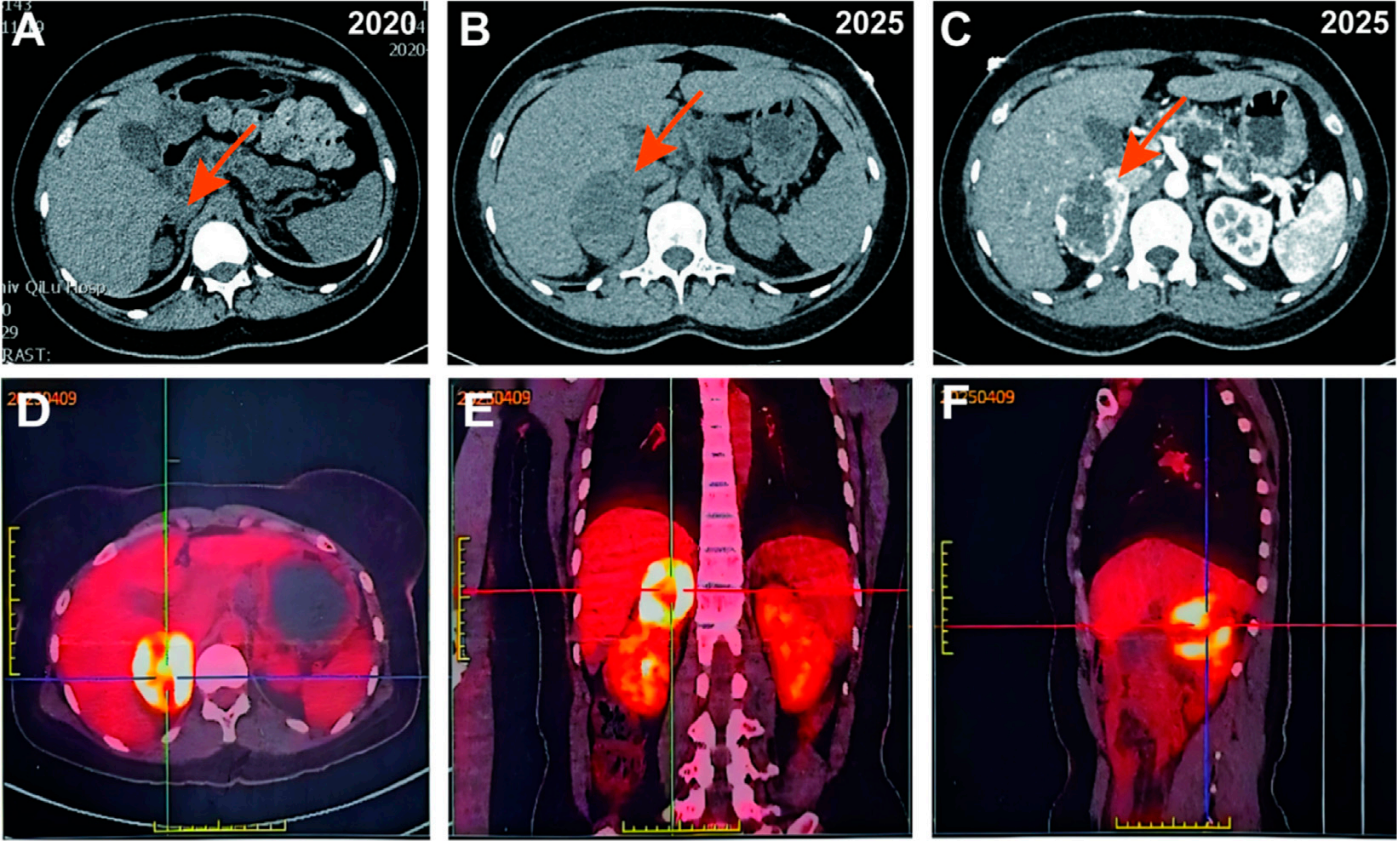
Adrenal gland imaging showing a space-occupying lesion in the left adrenal gland. **(A)** Adrenal glands in 2020. **(B,C)** Tumor of the right adrenal gland in 2025. **(D–F)** High-intensity FDG uptake of the mass in the right adrenal area in PET-CT.

Laboratory tests revealed that the hormone levels of aldosterone, renin and potassium were within normal ranges (Table 1). However, blood norepinephrine (31.58 nmol/L; reference range: 0–5.17) and methoxynorepinephrine (22.55 nmol/L; reference range: 0–0.17) were markedly elevated. The 18F-fluorodeoxyglucose (18F-FDG)-PET scan demonstrated a focal area of heterogeneous FDG uptake in the right adrenal region. Additionally, multiple cystic low-density lesions without significant FDG uptake were identified in the pancreas (Figure 2).

**TABLE 1:** Relevant laboratory tests and examinations for differentiating secondary hypertension.

Renin	50.89 pg/mL
Aldosterone	240.08 pg/mL
Triiodothyronine	3.72 pmol/L
Tetraiodothyronine	13.30 pmol/L
Thyroid stimulating hormone	3.360 µIU/mL
Potassium	4.28 mmol/L
Renal artery	The blood flow is unobstructed and no stenosis is observed

**FIGURE 3:**
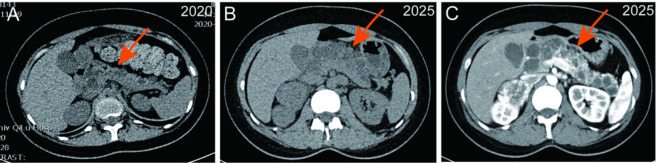
Pancreatic imaging showing multiple pancreatic cysts. **(A)** Pancreatic cysts in 2020. **(B,C)** Pancreatic cysts in 2025.

Following a multidisciplinary discussion, the right adrenal mass was highly suggestive of pheochromocytoma. Surgical intervention was recommended after preoperative preparation with oral phenoxybenzamine for 3 weeks. A retrospective review of imaging data from 4 years ago indicated substantial progression of the patient’s pancreatic cysts (Figure 3). Despite normal blood glucose levels, persistent hyperlipidemia (Low density lipoprotein cholesterol: 5.81 mmol/L; total cholesterol: 7.35 mmol/L) and elevated amylase and lipase levels were observed, suggesting impaired exocrine pancreatic function. Continued monitoring of the pancreatic condition is therefore advised. Given the patient’s young age and presence of multiple organ tumors, a hereditary tumor syndrome was suspected. Concurrently, genetic testing was performed to further investigate this possibility.

## Management (medical/interventions)

**FIGURE 4:**
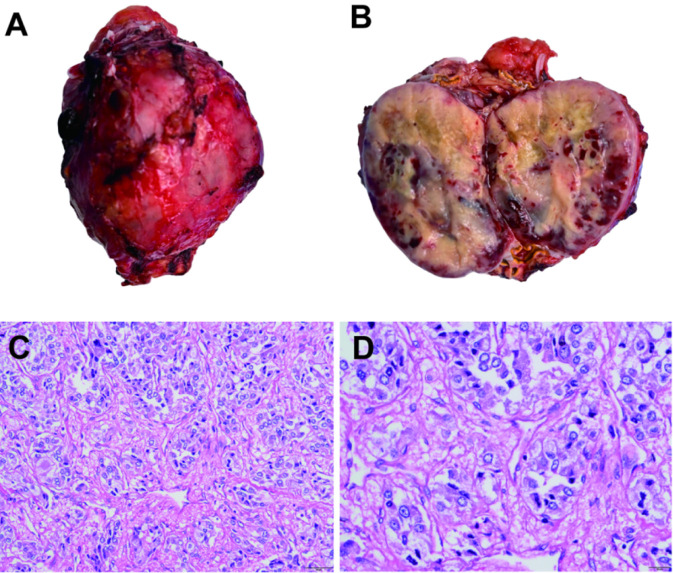
Adrenal mass imaging. **(A,B)** Gross image. **(C,D)** HE staining of tumor sections.

Three weeks later, the patient underwent laparoscopic right adrenalectomy, and postoperative pathological examination confirmed the diagnosis of pheochromocytoma (Figure 4). Histopathological and immunohistochemical staining results demonstrated that positive immunostaining for Syn, CgA, S-100, and SDHB were observed while SSTR2 and CK showed no positive staining, and Ki-67 labeling index was 3%, indicating low proliferative activity. Whole genome sequencing revealed a heterozygous deletion variant in exon 3 of the VHL gene (Supplementary Figure 1). According to the ACMG guidelines, this variant is preliminarily classified as pathogenic.

## Outcome and follow-up

The patient was followed up 1 month after the operation. Without the administration of antihypertensive medications, her blood pressure remained stable within the normal range, and visual acuity showed significant improvement compared to preoperative levels.

## Discussion

Based on the patient’s medical history, which includes spinal cord hemangioblastoma, pheochromocytoma, and multiple pancreatic cysts, along with the results of genetic testing, a diagnosis of von Hippel-Lindau disease was established. VHL disease is an autosomal dominant hereditary cancer disorder caused by pathogenic germline mutations in the VHL gene located on chromosome 3 (Latif et al., 1993). It has an estimated population prevalence of 1 in 47,000 individuals and affects 1 in 27,000 live births (Evans et al., 2010; Maher et al., 1991). Clinically, VHL disease is characterized by an increased predisposition to develop benign visceral cysts, most frequently affecting the pancreas and kidneys. Additionally, patients face a higher risk of developing tumors across multiple organ systems, typically exhibiting clear cell histology. Common clinical manifestations include retinal and central nervous system hemangioblastomas, clear cell renal cell carcinoma, pancreatic neuroendocrine tumors, pheochromocytoma/paraganglioma, as well as renal and pancreatic cysts. Less commonly observed features may include cysts of the epididymis or broad ligament and endolymphatic sac tumors (Shepherd et al., 2023). Most individuals with VHL disease begin to exhibit clinical symptoms after the age of 20, with a penetrance rate of 90%–100% by the ages of 65–70.

A clinical diagnosis of VHL disease can be established under the following conditions: the presence of at least two central nervous system hemangioblastomas (including retinal hemangioblastomas); the presence of at least one central nervous system hemangioblastoma and one of the other aforementioned manifestations; or the presence of any one of the aforementioned manifestations along with either a pathogenic mutation in the VHL gene or a first-degree familial relationship with an individual diagnosed with VHL disease (Chittiboina and Lonser, 2015).

The gold standard for diagnosing von Hippel-Lindau disease is the identification of a pathogenic variant in the VHL gene, which can confirm the clinical diagnosis. Although the clinical phenotypes exhibit significant heterogeneity, germline mutations in the VHL gene can be detected in nearly all affected individuals (Nielsen et al., 2016; Nordstrom-O'Brien et al., 2010). VHL disease is classified into two major clinical types based on the presence or absence of pheochromocytoma and renal cell carcinoma. Type 1 VHL is typically caused by loss-of-function mutations or those affecting protein folding. Truncated variants or exon deletions are frequently detectable and are associated with a lower risk of pheochromocytoma. In contrast, most cases of type 2 VHL are attributed to missense mutations. Type 2 VHL has been further categorized into subtypes: type 2A (associated with a lower risk of renal cell carcinoma), type 2B (associated with a higher risk of renal cell carcinoma), and type 2C (associated with isolated pheochromocytoma) (Ong et al., 2007; McNeill et al., 2009). Certain pathogenic variants may induce protein misfolding, thereby activating chaperone-mediated degradation pathways. Although this classification has advanced genotype-phenotype research, its clinical utility is increasingly limited due to the discovery of additional tumor types and the growing complexity of the disease. As illustrated in this case, the patient carried an exon deletion mutation and yet developed early-onset pheochromocytoma, leading to reclassification as type 2A (pheochromocytoma present without concurrent renal cell carcinoma). A retrospective study analyzed 31 patients with VHL syndrome complicated with pheochromocytoma and found that the exon 3 mutation was most common seen in 22 out of 31. Among them, three patients had large fragment deletions of the VHL gene, and all the three patients suffered from unilateral pheochromocytoma. Bilateral pheochromocytoma or multifocal pheochromocytoma/paraganglioma are significantly more common in patients with other mutations (Lomte et al., 2018). This appears to be inconsistent with the classical genotype-phenotype relationship. Various types of genetic variations can alter gene function and downstream signaling pathways, leading to phenotypic differences among individuals (Nielsen et al., 2016). However, the intrinsic relationships between diverse genotypes and phenotypes remain incompletely understood. This further indicates that for patients who have already undergone genetic testing, it is also very important to conduct comprehensive screening and follow-up.

Patients with VHL disease are predisposed to developing multiple types of pancreatic lesions, including parenchymal simple cysts, serous cystadenomas, and neuroendocrine tumors. Among these, simple pancreatic cysts are the most commonly observed. These cysts are typically benign and asymptomatic unless they cause compressive or obstructive symptoms. They tend to be diffusely distributed throughout the pancreas, and surgical intervention is generally indicated only when symptoms arise (Elli et al., 2006; Fernandes et al., 2022). In this patient, although diagnostic tests suggested impaired exocrine pancreatic function, there were no associated clinical manifestations. Moreover, the cystic changes were widespread, nearly encompassing the entire pancreas. Currently, there are no indications for surgery, and operative management does not appear to be the optimal treatment option at this stage.

Von Hippel-Lindau disease is inherited in an autosomal dominant manner. Affected individuals have a 50% chance of transmitting the mutation to each offspring. While most patients with VHL disease have a positive family history, some report no known familial occurrence of VHL-related tumors. This may be attributed to *de novo* or sporadic mutations, or mosaicism involving the VHL gene in one or both parents. In cases where the familial mutation is known, targeted genetic testing can be employed to identify asymptomatic at-risk relatives and facilitate presymptomatic screening for VHL-associated neoplasms, which offers significant clinical benefits to immediate family members. In this particular case, the patient denied any history of tumors in his parents. Despite the physician’s strong recommendation that the patient’s parents and daughter undergo genetic testing, the patient declined for various reasons. Although the diagnosis and treatment process for this patient has reached a temporary conclusion at this stage, long-term monitoring and follow-up remain essential. The VHL Family Alliance has established monitoring guidelines applicable to individuals diagnosed with VHL disease, those identified through presymptomatic testing, and high-risk family members of VHL patients who have not undergone molecular genetic testing. Annual eye examinations, blood pressure monitoring, and assessments of vision and hearing are recommended starting from age one. Beginning at age five, screening for pheochromocytoma via adrenaline level testing is advised. Abdominal ultrasound screening for visceral lesions should be initiated by age eight or earlier. Starting at age 16, abdominal ultrasounds should be conducted at least every 2 years to evaluate the kidneys, pancreas, and adrenal glands. In addition, MRI scans of the brain and entire spine are recommended for the early detection of central nervous system lesions.

## Conclusion

Although VHL disease is a rare condition, it warrants significant clinical attention in patients presenting with hypertension and multiple organ neoplasms. Comprehensive evaluation, standardized familial screening, and genetic testing play a crucial role in guiding treatment decisions for affected individuals. Due to the presence of comorbidities, the inherent complexity of the disease, and the need for ongoing monitoring of small asymptomatic lesions to detect signs of progression, patients with VHL disease require long-term medical attention and surveillance.

## Take home messages

1. Von Hippel-Lindau (VHL) disease is an autosomal dominant hereditary cancer syndrome characterized by a wide range of clinical phenotypes, which frequently affect the central nervous system, kidneys, adrenal glands, and pancreas.
2. The gold standard for diagnosing VHL disease is the identification of pathogenic variants in the VHL gene. Germline mutations in the VHL gene can be detected in nearly all individuals diagnosed with VHL disease.

## Data Availability

The raw data supporting the conclusions of this article will be made available by the authors, without undue reservation.

## References

[B1] Chittiboina P. Lonser R. R. (2015). Von Hippel-Lindau disease. Handb. Clin. Neurol. 132, 139–156. 10.1016/B978-0-444-62702-5.00010-X 26564077 PMC5121930

[B2] Elli L. Buscarini E. Portugalli V. Reduzzi L. Reduzzi C. Brambilla G. (2006). Pancreatic involvement in von Hippel-Lindau disease: report of two cases and review of the literature. Am. J. Gastroenterol. 101, 2655–2658. 10.1111/j.1572-0241.2006.00737.x 16952288

[B3] Evans D. G. Howard E. Giblin C. Clancy T. Spencer H. Huson S. M. (2010). Birth incidence and prevalence of tumor-prone syndromes: estimates from a UK family genetic register service. Am. J. Med. Genet. A 152a, 327–332. 10.1002/ajmg.a.33139 20082463

[B4] Fernandes D. A. Mourão J. L. V. Duarte J. Dalaqua M. Reis F. Caserta N. M. G. (2022). Imaging manifestations of von Hippel-Lindau disease: an illustrated guide focusing on abdominal manifestations. Radiol. Bras. 55, 317–323. 10.1590/0100-3984.2021.0121-en 36320367 PMC9620840

[B5] Latif F. Tory K. Gnarra J. Yao M. Duh F. M. Orcutt M. L. (1993). Identification of the von Hippel-Lindau disease tumor suppressor gene. Science 260, 1317–1320. 10.1126/science.8493574 8493574

[B6] Lomte N. Kumar S. Sarathi V. Pandit R. Goroshi M. Jadhav S. (2018). Genotype phenotype correlation in Asian Indian von Hippel-Lindau (VHL) syndrome patients with pheochromocytoma/paraganglioma. Fam. Cancer 17, 441–449. 10.1007/s10689-017-0058-y 29124493

[B7] Maher E. R. Iselius L. Yates J. R. Littler M. Benjamin C. Harris R. (1991). Von Hippel-Lindau disease: a genetic study. J. Med. Genet. 28, 443–447. 10.1136/jmg.28.7.443 1895313 PMC1016952

[B8] McNeill A. Rattenberry E. Barber R. Killick P. MacDonald F. Maher E. R. (2009). Genotype-phenotype correlations in VHL exon deletions. Am. J. Med. Genet. A 149a, 2147–2151. 10.1002/ajmg.a.33023 19764026

[B9] Nielsen S. M. Rhodes L. Blanco I. Chung W. K. Eng C. Maher E. R. (2016). Von Hippel-Lindau Disease: genetics and Role of Genetic Counseling in a Multiple Neoplasia Syndrome. J. Clin. Oncol. 34, 2172–2181. 10.1200/JCO.2015.65.6140 27114602

[B10] Nordstrom-O'Brien M. van der Luijt R. B. van Rooijen E. van den Ouweland A. M. Majoor-Krakauer D. F. Lolkema M. P. (2010). Genetic analysis of von Hippel-Lindau disease. Hum. Mutat. 31, 521–537. 10.1002/humu.21219 20151405

[B11] Ong K. R. Woodward E. R. Killick P. Lim C. Macdonald F. Maher E. R. (2007). Genotype-phenotype correlations in von Hippel-Lindau disease. Hum. Mutat. 28, 143–149. 10.1002/humu.20385 17024664

[B12] Shepherd S. T. C. Drake W. M. Turajlic S. (2023). The road to systemic therapy in von Hippel-Lindau (VHL) disease: are we there yet? Eur. J. Cancer 182, 15–22. 10.1016/j.ejca.2022.12.011 36708612

