# Supplementary figures and images for: Case Report: von Hippel-Lindau (VHL) disease: a young female presenting with multiple organ tumors
Source: Front Genet. 2025 Oct 17;16:1676039. doi: 10.3389/fgene.2025.1676039 (PMC12574975; doi:10.3389/fgene.2025.1676039)

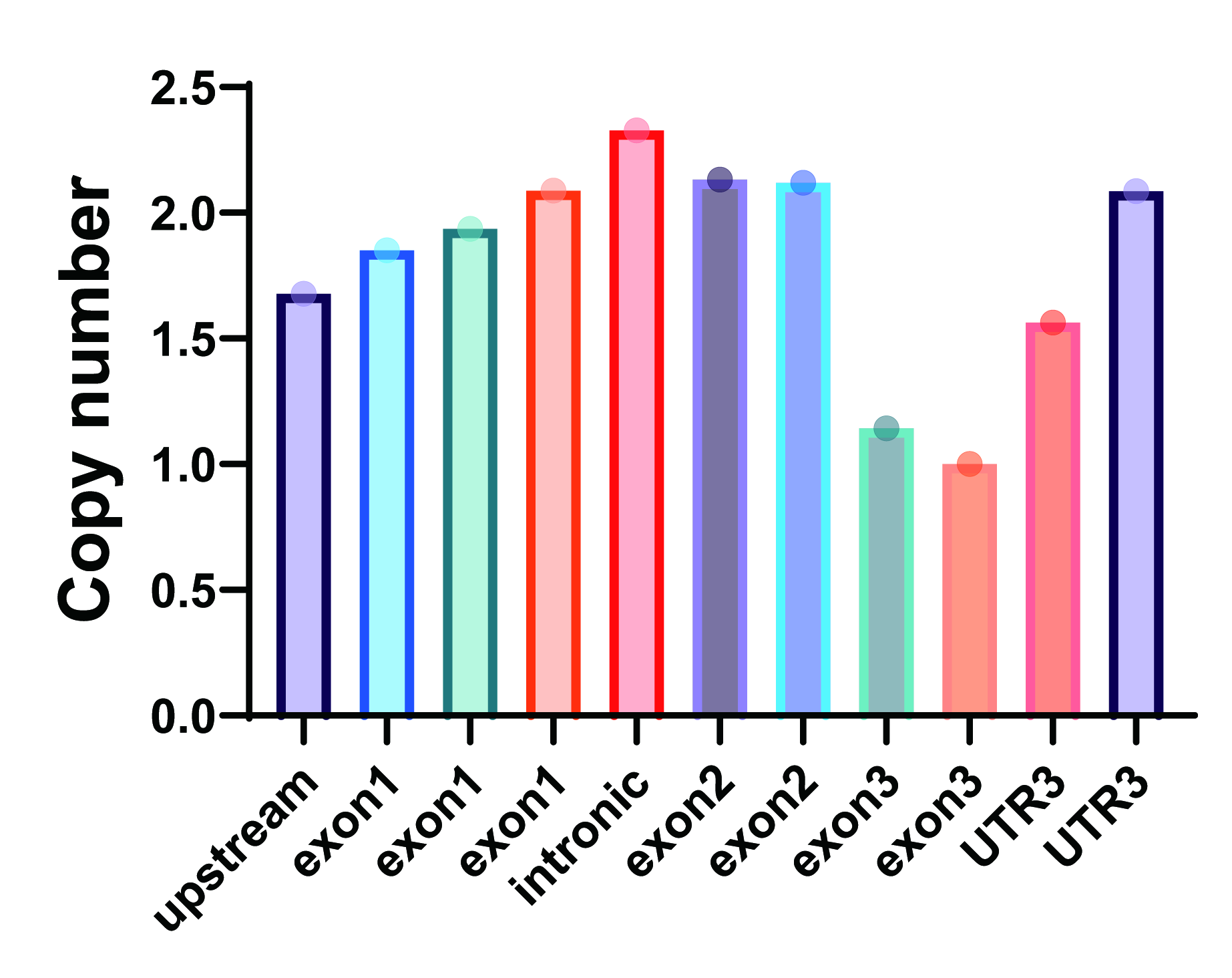

Supplement: Supplementary file 1 [file Image1.tif]
